# Diagnostic yield and renal complications after computed tomography pulmonary angiograms performed in a community-based academic hospital

**DOI:** 10.3402/jchimp.v2i2.17722

**Published:** 2012-07-16

**Authors:** Zacharia Reagle, Steven Tringali, Narinder Gill, Michael W. Peterson

**Affiliations:** UCSF Fresno Department of Medicine, Fresno, CA, USA

**Keywords:** venous thromboembolism, pulmonary embolism, computed tomography pulmonary angiogram, contrast induced nephropathy

## Abstract

**Background:**

Venous thromboembolism and pulmonary embolism (VTE/PE) remain a diagnostic challenge. The computed tomography pulmonary angiogram (CTPA) has emerged as a popular diagnostic test for PE. However, there is limited data on diagnostic yield and complications in actual clinical settings. Our goal was to determine the diagnostic yield for PE and rate of renal complications following CTPA in a large community hospital setting.

**Methods:**

A retrospective chart review of 1,514 patients who underwent CTPA in the emergency department or during the initial 24 hours of admission to a community-based academic hospital.

**Results:**

Of 1,514 CTPAs, 125 were positive for VTE/PE yielding a positive diagnosis in 8.2%. Dyspnea was the most common symptom in patients and a normal physical exam was the most common finding. Among the 925 patients with adequate data to calculate the rate of contrast-induced nephropathy (CIN), 25.8% had an increase of at least 25% in serum creatinine following the CTPA. Pre-existing diabetes and age were the most important predictors of CIN.

**Conclusions:**

CTPA has a low diagnostic yield for PE in a community setting, and in some patient populations, the rate of contrast-induced nephropathy may be higher than previously reported in the literature. Due to the retrospective nature of this study we were limited in using pre-test scoring systems and in measuring the impact of alternative CT diagnoses on patient management.

Venous thromboembolic disease including pulmonary embolism (VTE/PE) is a life-threatening systemic disease with varied clinical presentation. The clinical presentation of VTE/PE can be non-specific and the potential mortality of an undiagnosed PE is concerning (1). This combination of a potentially lethal disease presenting with non-specific symptoms has generated a great deal of research into imaging technologies and clinical decision-making in this patient population. While pulmonary angiography has been the historic gold standard for diagnostic imaging, its use is limited by the required expertise and its invasive nature (2). The Ventilation–Perfusion scan (V/Q scan) has been the most widely used imaging study for the diagnosis of VTE/PE. In the clinical setting, V/Q scans are limited by baseline radiographic abnormalities in the patients and by the frequency of intermediate probability scans (3).

In recent years, the multi-detector computed tomography pulmonary angiogram (CTPA) has become available and has quickly replaced V/Q scanning as the clinicians’ imaging study of choice for the diagnosis of VTE/PE (4–6). The CTPA is an attractive study for most clinicians because it is widely available in emergency departments, provides a clear positive or negative diagnosis of PE and may detect other pulmonary pathology that a V/Q scan or pulmonary angiography would not diagnose (4). Over the last decade the utilization of CTPA has rapidly proliferated with increases ranging from 227% to over 13-fold (5–8). This rapid proliferation has occurred with limited evaluation of outcomes or risk. As with many new technologies, the original studies were performed under very stringent clinical research control, but little research has evaluated risk and benefit in actual clinical conditions.

CTPA is performed using a helical CT scan. Between 100 and 160 ml of low osmolar contrast material is injected in a bolus and timed to optimize visualization of the contrast in the pulmonary arterial circulation. Compared with coronary angiograms (contrast load of 30–50 ml), this is a large contrast dose. While the rate of contrast nephropathy from coronary angiograms has been reported, the rate of contrast nephropathy from CTPAs has not been well studied.

In addition to possible risks of contrast exposure, CTPA's also provide risk from radiation exposure. Currently the actual radiation induced malignancy risk is not well understood, however recent literature has suggested that it may be higher than previously thought (9, 10).

Because of the limited data about diagnostic yield for PE of CTPA's in non-research settings and the risks attendant to them, we designed our study to investigate the diagnostic yield and risks when used in a community hospital setting.

## Methods

We performed a retrospective chart review of all CTPA's either performed in the emergency department or during the initial 24 hours of hospitalization in our hospital for a 12-month period from 1 January 2008 to 31 December 2008. Our hospital, Community Regional Medical Center, is a 600 bed community-based academic hospital that is affiliated with the University of California San Francisco School of Medicine. It is a regional referral center and level one trauma center with a mixed academic and private medical staff serving the diverse population of California's San Joaquin Valley. CTPA's were performed using a 64 slice multi-detector machine made by General Electric, Fairfield, Connecticut.

We collected demographic data including age, gender, ethnicity, and occupation on our patients. In addition, we abstracted the patient record for any history of previous VTE/PE, recent surgery or fracture, malignancy, or pregnancy, as well as characteristic symptoms of VTE/PE including the presence of hemoptysis, dyspnea, or lower extremity pain or edema. When available, we collected serum creatinine prior to the CTPA and serum creatinine 48 hours after the CTPA. We abstracted the radiology reports for evidence of pathology on plain films and on chest CT scans in addition to the presence or absence of pulmonary embolism.

We excluded any patients already taking therapeutic anticoagulation from our study. We also excluded all patients with end-stage renal disease on hemodialysis during the analysis of contrast-induced nephropathy.

Our study was reviewed and approved by the Institutional Review Board of Community Medical Centers.

## Results

### Diagnostic yield

During our study period 1,514 CTPA's meeting our inclusion criteria were performed. Table 1 presents the demographic information on our patient population. The ethnic composition of our study group reflects our general patient population.

**Table 1:** Patient baseline characteristics

Characteristic	All patients
Age – yr (mean±SD)	53.1±17.9
Male gender – no. (%)	610 (40.3)
Female gender	904 (59.7)
Ethnicity – no. (%)	
Hispanic	664 (43.8)
Caucasian, non-Hispanic	540 (35.6)
African American	214 (14.1)
Asian	88 (5.8)
Other	7 (0.5)
Unknown	2 (0.1)
Symptoms – no. (%)	
Hemoptysis	39 (2.6)
Leg pain/edema	234 (15.5)
Dyspnea	1390 (91.8)
Prior history – no. (%)	
DVT/PE	107 (7.1)
Surgery	63 (4.2)
Recent fracture	14 (0.9)
Malignancy	100 (6.6)
Pregnancy	31 (2.1)
Lung exam findings – no. (%)	
Normal	1087 (71.8)
Crackles	198 (13.1)
Wheezes	136 (9)
Stridor	10 (0.7)
Crackles and wheezes	79 (5.2)
Missing data	4 (0.3)
CXR prior to CTPA – no. (%)	
Multiple findings	670 (44.3)
Normal	640 (42.3)
Other	122 (8.1)
None ordered	70 (4.6)
Cardiomegaly	4 (0.3)
Infiltrate	3 (0.2)
Pulm congestion	2 (0.1)
Consolidation	2 (0.1)
Scarring	1 (0.1)
Serum creatinine – mg/dl (mean±SD)	1.1±1.7

DVT/PE, deep venous thrombosis/pulmonary embolism; CXR, chest X-ray; CTPA, computed tomography pulmonary angiogram.

As shown in the table, the most common presenting symptom was dyspnea, which was present in 91.8% of patients. Hemoptysis was an uncommon symptom occurring in only 2.6% of patients. A history of prior VTE/PE was present in 7.1% of patients. On physical exam, the most common finding was a normal lung exam, which was reported in 71.8% of patients.

Of the 1,514 CTPA's, 125 of them were positive for VTE/PE, which is a positive diagnostic yield of 8.2%. The positive diagnostic yield did not differ by hospital location or specialty service that ordered the test (Table 2).

**Table 2:** Diagnostic yield by ordering service

Finding	Emergency department (*N*=1139)	Academic internal medicine (*N*=133)	Private internal medicine (*N*=241)	*p*-Value
Pulmonary embolism – *n* (%)	95 (8.34)	11 (8.27)	19 (7.88)	0.973

CTPA, computed tomography pulmonary angiogram.

There are two clinical scoring systems currently in clinical use, the Well's score and the Revised Geneva score (11, 12). These scoring systems help define pre-test probability. It is not possible to retroactively apply the Well's score because the assessing physician's clinical judgment is a significant component of the Well's score. However the Revised Geneva score uses strictly objective data and therefore this score can be retrospectively calculated.

Using the Revised Geneva scoring system, the patients are grouped into one of three categories: Low probability, Intermediate probability or High probability. Using this scoring system, 447 CTPAs (29.5%) had a Low Geneva score. Of the CTPAs performed in patients with a Low Geneva score, 20 (4.5%) were positive for pulmonary embolism. One thousand and twelve patients in our study (66.8%) had an intermediate score. Of those, 91 (9.0%) were positive for a pulmonary embolism. The 55 remaining patients had a calculated Revised Geneva score of High probability. Of those, 14 (25.4%) were positive for a pulmonary embolism (Table 3).

**Table 3:** Diagnostic yield by Geneva score

Finding	Geneva score = 1 (*N*=447)	Geneva score = 2 (*N*=1012)	Geneva score = 3 (*N*=55)	*p*-Value
Pulmonary embolism – *n* (%)	20 (4.5)	91 (9)	14 (25.5)	<0.0001*

CTPA, computed tomography pulmonary angiogram; PE, pulmonary embolism.

* Pairwise comparison between all categories were significant (*p*<0.005).

### Complications and risk

Of the 1,514 patients in our study, 39 had pre-existing end-stage renal disease (ESRD) and were on hemodialysis at the time of the CTPA. Those 39 patients were removed from evaluation for potential contrast induced nephropathy (CIN). Of the remaining 1475 patients, 34 of them had no pre-CTPA creatinine and 516 of them had no 48-hour post-CTPA creatinine. Most of the patients with no 48-hour post-CTPA creatinine were either discharged from the emergency room or admitted to the hospital for less than 48 hours. The remaining 925 patients had both a pre-CTPA creatinine and a 48-hour post-CTPA creatinine. Of those 925 patients, 239 of them had an increase in creatinine of > 25% for a rate of CIN of 25.8%. None of the patients required renal replacement therapy.

Table 4 reports the characteristics of patients who experienced contrast-induced nephropathy. Age and pre-existing diabetes were the most important risk factors for CIN.

**Table 4:** Characteristics of patients with and without contrast-induced nephropathy

Characteristic	Patient with CIN (*N*=239)	Patients without CIN (*N*=686)	*p*-Value
Age – yr	60.1±16.3	55.8±17.6	<0.001
Male gender – no. (%)	100 (41.8)	300 (43.7)	0.61
Ethnicity – no. (%)			0.53
Caucasian, non-Hispanic	97 (40.6)	275 (40.1)	
Hispanic	94 (39.3)	267 (38.9)	
African American	36 (15.1)	88 (12.8)	
Asian	11 (4.6)	53 (7.7)	
Other/unknown	1 (0.4)	3 (0.4)	
Diabetes	79 (33.1)	1 (0.1)	<0.001
BMI – kg/m^2^	31.2±10.2	31±18	0.74
Serum Creatinine – mg/dl	0.9±1	1.1±1.8	0.06

CIN, contrast induced nephropathy; BMI, body mass index.

## Discussion

The diagnostic yield of CTPAs performed in our facility within 24 hours of presentation to the emergency department was 8.2%. This positive diagnostic yield needs to be interpreted in the setting of the cost and risks associated with CTPA. Our diagnostic yield is not unusual and is supported by previous studies reporting positive studies for PE from 5.7 to 9.0% (5–8, 13).

One approach previously suggested to increase the diagnostic yield of CTPA's is clinical scoring systems to define pretest probability. The landmark PIOPED II study reported the importance of a pretest scoring system (12, 13) and appropriate use of the D-dimer test as a screening tool prior to imaging studies in the diagnostic evaluation of VTE/PE (4). Unfortunately, D-dimer tests were infrequently ordered in our population prior to the CTPA. Due to the retrospective nature of our study and the large number of physicians with varying degrees of clinical expertise and background, we do not know if such scoring systems were routinely applied. However the low utilization of D-dimers suggests a low level of algorithm use. When we applied the only scoring system with completely objective data, the Geneva score, retrospectively, we found discriminatory power in the intermediate and high risk groups. However, in the low risk group, more patients were diagnosed with PE than the Geneva score would predict. To the best of our knowledge, pretest scoring systems, such as the Geneva score, have not previously been evaluated outside of clinical research protocols. Therefore one possible explanation for the higher than expected rate of VTE/PE in the Geneva score low risk group is that this scoring system loses some of it's discriminatory power when used outside of clinical research protocols. Another possible explanation could be overdiagnosis by CTPA in the low risk group. Our facility uses a 64 slice multi-detector CT scan. This is a highly sensitive detector and has the ability to detect disease in very small vessels. Clots in these small vessels may be of questionable clinical significance (3, 8, 14).

Supporting this potential explanation, Burge and colleagues preformed an epidemiologic review of the diagnosis of PE and PE mortality in the state of New York from 1 January 1994 to 31 December 2004. During this study period, the imaging technique of choice changed from ventilation/perfusion (V/Q) scan to CTPA. During this time they found an almost doubling of the diagnosis of PE. One would expect that an increase in the diagnosis and subsequent treatment would lead to a reduction in mortality; however, this did not occur. The mortality rate from PE did not vary over the same 11-year period. The authors felt these data raised a serious question about the clinical significance of the increased diagnostic rate and questioned the appropriateness of patient selection for CTPA in suspected PE (14). The authors concluded that providers should consider the cost, radiation exposure, and possibility of unnecessary anti-coagulation in a risk-benefit analysis prior to ordering a CTPA during the evaluation of suspected PE.

### Harm or injury associated with CTPA

Our data also demonstrate a high incidence of contrast-induced nephropathy. In general CIN is associated with age > 75, diabetes, non-steroidal anti-inflammatory use and multiple myeloma (15–17). For purposes of our study, we defined CIN as an increase in the baseline creatinine of at least 25%. This definition of CIN remains the most commonly used definition in randomized control trials (16–18). This definition seems clinically reasonable because rises in creatinine as low as 25% are associated with increased mortality rates (19, 20). Using that definition, our incidence of CIN was 25.8%. Previous smaller studies have reported CIN rates after CTPA of 8.9–12% (15, 21).

Among our patients, the most significant risk factor for CIN was a history of diabetes. Of the 80 patients with diabetes who underwent a CTPA and had both a pre-CTPA and 48-hour post-CTPA creatinine, 79 (98.8%) experienced CIN. The relative risk of developing CIN in diabetics was 5.22. Unlike prior descriptions of pre-existing renal insufficiency as a risk factor for diabetics to develop CIN, only 2 of the 79 patients with diabetes who experienced CIN had a pre-CTPA creatinine greater than 1.5 mg/dl (22). The high incidence of diabetes in our general patient population may have contributed to our higher incidence of CIN. Consistent with earlier studies, we also found that increased age was an independent predictor of CIN. Patients with CIN were on average 5 years older than those without CIN.

Our study was not designed to address the additional risk associated with radiation exposure. However, future studies should address potential cancer risk linked to radiation exposure.

Our study has some limitations. Due to the retrospective nature of our study we do not know what clinical question the ordering physician was attempting to answer. It is possible that our low diagnostic yield could be due in part to physicians ordering the CTPA as a ‘catch-all’ test for any pulmonary pathology. Furthermore we also were not able to evaluate the harms associated with radiation exposure. There is growing evidence in the literature that cohorts of patients exposed to medical radiation have an associated risk for the development of radiation-induced cancers. (9, 22–26).

## Conclusions

The positive diagnostic yield for PE by CTPA's performed in the emergency room or during the initial 24 hours of hospitalization in our community-based academic hospital was 8.2%. This diagnostic yield was similar across ordering physicians’ specialty. The rate of CIN was 25.8%. Organizational steps to try to improve the diagnostic yield and reduce the incidence of CIN are warranted.
